# Supplementary material for: High-κ van der Waals Oxide MoO3 as Efficient Gate Dielectric for MoS2 Field-Effect Transistors
Source: Materials (Basel). 2022 Aug 25;15(17):5859. doi: 10.3390/ma15175859 (PMC9457482; doi:10.3390/ma15175859)
Supplement: Supplementary file 1 [file materials-15-05859-s001.zip › materials-1853027-supplementary.pdf]

Supporting Information

High-κ van der Waals Oxide MoO_3 as Efficient Gate Dielectric for MoS_2 Field-Effect Transistors

Growth of MoO_3 Nanosheets

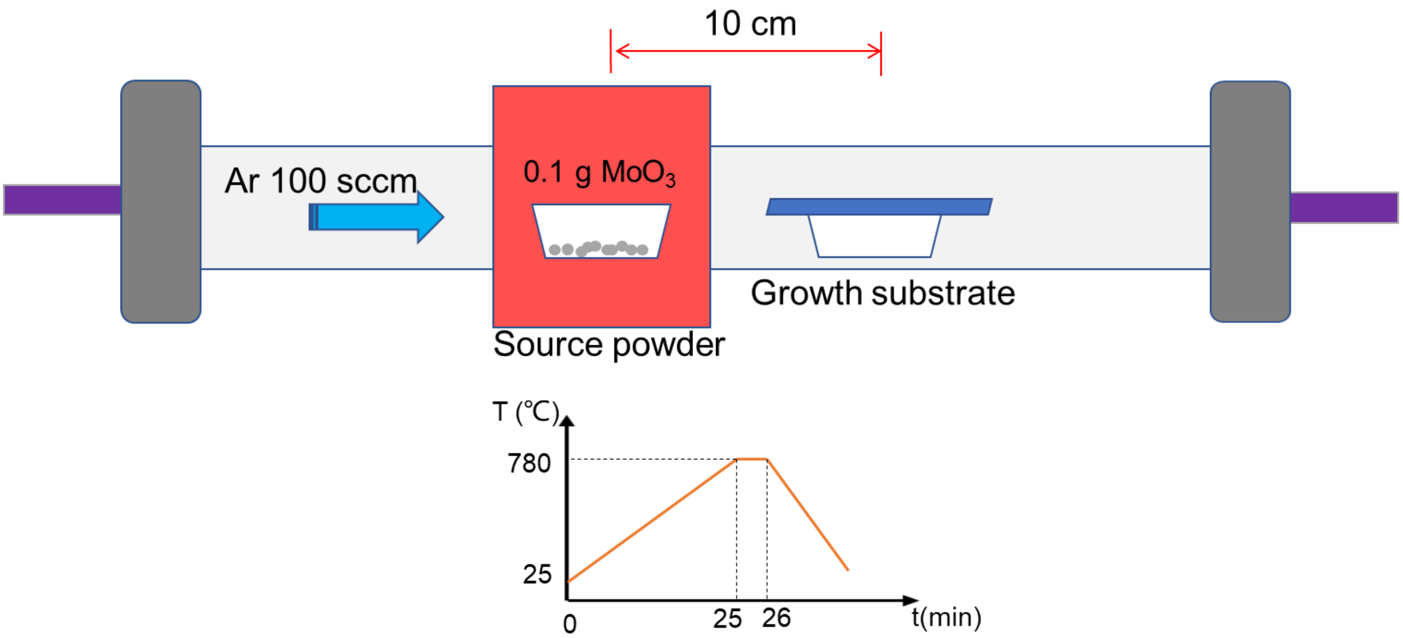

Figure S1. The detailed process of growing MoO_3 nanosheets using vapor deposition method, the resulting nanosheets are shown in Figure 1b.

Leakage Current of MoO_3

Good insulation is the key to gate dielectric applications. As shown in Figure S2, the MoO_3 FET device presented a high resistance state of 10^{-13} A, which was approximately two orders of magnitude smaller than the smallest current measured while gating the device, suggesting the good insulation of MoO_3.

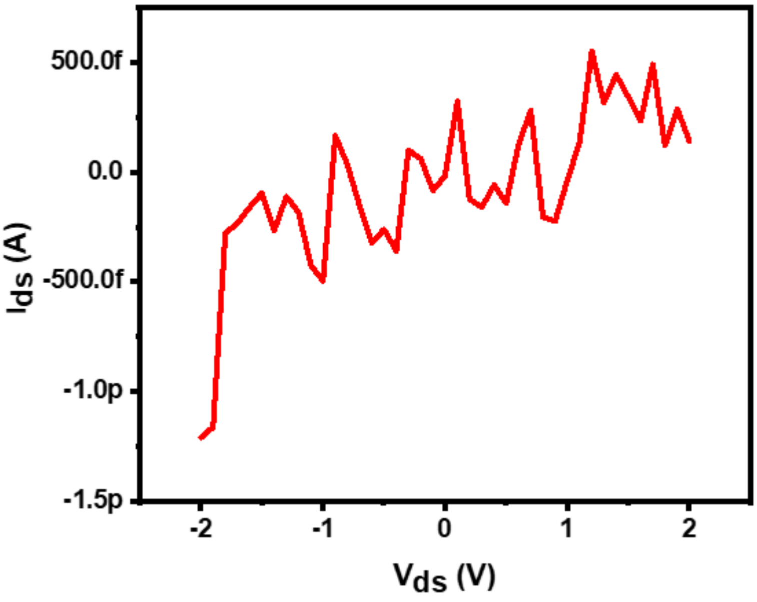

Figure S2. I–V curve of MoO_3.

Gr/MoO_3/MoS_2 Heterostructure Memory Devices Fabrication

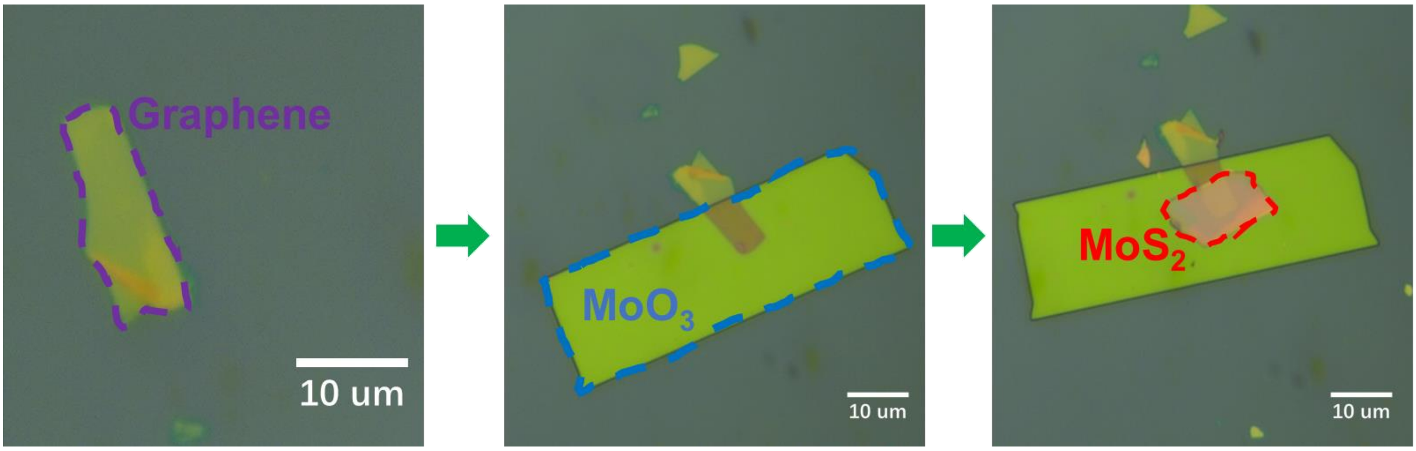

Figure S3. Gr/MoO_3/MoS_2 heterostructure transfer process.
